# Biaxial Flexural Strength of Different Monolithic Zirconia upon Post-Sintering Processes

**DOI:** 10.1055/s-0041-1735937

**Published:** 2022-01-11

**Authors:** Niwut Juntavee, Apa Juntavee, Thipradi Phattharasophachai

**Affiliations:** 1 Department of Prosthodontics, Faculty of Dentistry, Khon Kaen University, Khon Kaen, Thailand; 2 Department of Preventive Dentistry, Division of Pediatric Dentistry, Faculty of Dentistry, Khon Kaen University, Khon Kaen, Thailand; 3 Division of Biomaterials and Prosthodontics Research, Faculty of Dentistry, Khon Kaen University, Khon Kaen, Thailand

**Keywords:** flexural strength, glazing, heat treatment, polishing, post-sintering process, zirconia

## Abstract

**Objective** Different post-sintering processes are expected to be a reason for alteration in the strength of zirconia. This study evaluated the effect of post-sintering processes on the flexural strength of different types of monolithic zirconia.

**Materials and Methods** A total of 120 classical- (Cz) and high-translucent (Hz) monolithic zirconia discs (1.2 mm thickness and 14 mm in Ø) were prepared, sintered, and randomly divided into four groups to be surface-treated with (1) as-glazed (AG); (2) finished and polished (FP); (3) finished, polished, and overglazed (FPOG); and (4) finished, polished, and heat-treated (FPHT) technique ( *n* = 15). Biaxial flexural strength (σ) was determined on a piston-on-three ball in a universal testing machine at a speed of 0.5 mm/min.

**Statistical Analysis** Analysis of variance, and post hoc Bonferroni multiple comparisons were determined for significant differences ( *α = 0.05* ). Weibull analysis was applied for survival probability, Weibull modulus (m), and characteristic strength (σ _0_ ). The microstructures were examined with a scanning electron microscope and X-ray diffraction.

**Results** The mean ± standard deviation value of σ (MPa), m, and σ _0_ were 1,626.43 ± 184.38, 9.51, and 1,709.79 for CzAG; 1,734.98 ± 136.15, 12.83, and 1,799.17 for CzFP; 1,636.92 ± 130.11, 14.66, and 1,697.63 for CzFPOG; and 1,590.78 ± 161.74, 10.13, and 1,663.82 for CzFPHT; 643.30 ± 118.59, 5.59, and 695.55 for HzAG; 671.52 ± 96.77, 3.28, and 782.61 for HzFP; 556.33 ± 122.85, 4.76, and 607.01 for HzFPOG; and 598.36 ± 57.96, 11.22, and 624.89 for HzFPHT. The σ was significantly affected by the post-sintering process and type of zirconia ( *p* < 0.05), but not by their interactions ( *p* > 0.05). The Cz indicated a significantly higher σ than Hz. The FP process significantly enhanced σ more than other treatment procedures.

**Conclusion** Post-sintering processes enabled an alteration in σ of zirconia. FP enhanced σ, while FPOG and FPHT resulted in a reduction of σ. Glazing tends to induce defects at the glazing interface, while heat treatment induces a phase change to tetragonal, both resulted in reducing σ. Finishing and polishing for both Cz and Hz monolithic zirconia is recommended, while overglazed or heat-treated is not suggested.

## Introduction

Nowadays, all-ceramic restoration has become popular and plays an important role in contemporary restorative dentistry, which is capable of providing a natural esthetic restoration. The ceramic materials must possess high esthetics and be fracture-resistant, especially in the load-bearing area. 1 2 Zirconia has been using as a substructure for fixed prosthesis owing to its strength and white color. Zirconia is an inert white crystalline oxide of zirconium and possesses high biocompatibility. 3 It comprises three crystalline phases: monoclinic (m), tetragonal (t), and cubic (c). The m-phase is stable at room temperature, turns to t-phase beyond 1,170°C, and changes to c-phase at 2,370°C. The m-phase is not a strong crystalline structure, compared to the t-phase. 4 Thus, the t-phase is necessary and it can be stabilized at room temperature by adding stabilizing oxides such as 3% mol. of yttrium-oxide (Y _2_ O _3_ ) particles, resulting in a 3-yttrium partially stabilized tetragonal zirconia polycrystal (3Y-TZP). When the material is subjected to surface stress and subsequent cracks, high compressive stress can be developed at the crack tips, leading to t- → m-phases transformation with 4 to 4.5% volumetric expansion, rendering crack inhibition phenomenon, known as transformation toughening. 1 4 The stress can be generated from the temperature change or surface grinding, which eventually induces superficial modifications, damage, crack propagation, premature aging, and phase transformation. 5 The primitive zirconia is quite an opacity and needs to be veneered with porcelain to achieve a natural-looking appearance. However, the most common complication of porcelain-veneering zirconia is porcelain delamination. The classical translucence monolithic 3Y-TZP has been developed to eliminate the opaqueness of conventional zirconia. The restoration can be fabricated with the reduced amount of tooth preparation and restoration thickness, to be as less as 0.5 to 0.7 mm. 3 6 The translucency of zirconia is also achieved by increasing the sintering temperature, reducing alumina, or increasing the amount of Y _2_ O _3_ . Adding 5% mol. of Y _2_ O _3_ yields a high amount of cubic (c) phase with a smaller grain size of 5-yttrium partially stabilized zirconia (5Y-PSZ). It shows the best enhancement of translucency and aging resistance over the classical 3Y-TZP. 7 8 The 5Y-PSZ comprises fewer t-phase that exhibit less stress-induced phase transformation and less strength enhancement compared to classical 3Y-TZP. 8 9 10 11

Post-sintering processes are clinical procedures that clinicians need to perform on the zirconia restorations before delivery to the patients. The restorations need to be ground, adjusted, finished, polished, glazed, or heat-treated. 12 13 14 15 16 The diamond bur of grit size number >100 is usually used for grinding, though restoration is nearly perfect after sintering. 12 Both the intaglio and occlusal surfaces must be adjusted clinically for a better fit of the restoration. 13 14 15 16 It is found that a high-speed handpiece with water cooling produces less heat than a micromotor, but there is no significant difference in flexural strength between these tools or between the continuous and intermittent grinding methods. Grinding zirconia causes two counteractions: crack healing due to compressive stress-induced transformation toughening; and microcracks, which form deep surface flaws over the compression. 16 Although the grinding affects the flexural strength, appropriate polishing is required to smooth the roughened surface. 5 12 13 14 15 16 17 18 19 The advantages of polished surface include the prevention of plaque accumulation, wear reduction of opposing natural teeth, and maintenance of flexural strength, as well as a lower m-phase after aging. 19 20 The shiny, glossy surface of polished zirconia might be comparable to glazed zirconia. 2 21 The glazing process comprises a thin layer of glass covering the external surface of the restoration to improve its esthetics and roughness. Occasionally, staining and glazing are carried out after surface adjustment because finishing and polishing procedures remove the glazed layer and external stains that affect the color of zirconia. 10 22 Heat treatment is a process that aims to release a compressive layer, reverse the damage from the grinding procedure, and reduce the m-phase, which harms the long-term performance of zirconia. The heat treatment protocol includes variations in temperature and time using a ceramic furnace. There is evidence of a greater smoothness in the material surface upon heat treatment at 850°C for 1 minute. 17 23 The firing cycle upon staining and glazing can also act as a heat treatment that is capable of reduction in the m-phase, suggesting that the glazing can be used as a process to reverse the t-→m-phase transformation. 17 24 There is no standard protocol for monolithic zirconia adjustment after sintering. The controversy exists regarding the strengthening effects of clinical adjustment by grinding with burs, polishing, glazing, or heat treatment for the restoration. 14 21 As such, this study aimed to investigate the biaxial flexural strength of different types of zirconia upon various post-sintering processes. The null hypothesis was that glazing, grinding and polishing, overglazing after polishing, and heat treatment after polishing would not affect the biaxial flexural strength of different types of monolithic zirconia.

## Materials and Methods

### Preparation of Zirconia Specimens

The zirconia blanks were milled into a cylindrical shape with an 18 mm diameter (Ø) from precolored classical translucent monolithic zirconia (Cz, BruxZir Shaded, Prismatik, Irvine, California, United States), and high translucent monolithic zirconia (Hz, BruxZir Anterior shaded) and sectioned into a disc shape of 1.6 mm in thickness by using a sectioning machine (Isomet 1000, Beuhler, Lake Buff, Illinois, United States). The dimension of zirconia discs was compensated for sintering shrinkage with the enlargement factor of 1.2302 for Cz and 1.2334 for Hz. The discs were ground flat on both surfaces by silicon carbide abrasive paper with grit 500, 800, and 1,200, respectively, with water coolant on a polishing machine (Ecomet 3, Buehler) at a speed of 300 rounds per minute (rpm). Then, the specimens were sintered in a furnace (inFire HTC, Sirona, Bensheim, Germany), following the manufacturer's recommendation. A total of 120 zirconia discs of thickness (1.2 ± 0.2 mm) and Ø (14 ± 0.2 mm) were derived. The zirconia discs were sandblasted with 50 µm of alumina oxide powder with a pressure of 30 psi. The specimens were cleaned and allowed to dry at room temperature. The mixing of glazing paste and liquid (IPS e.max Ceram, Ivoclar Vivadent, Schaan, Liechtenstein) was applied over the blasted surface and fired in a ceramic furnace (Programat P-310, Ivoclar Vivadent) to produce a glazed surface.

### Post-Sintering Surface Treatment

**Fig. 1:**
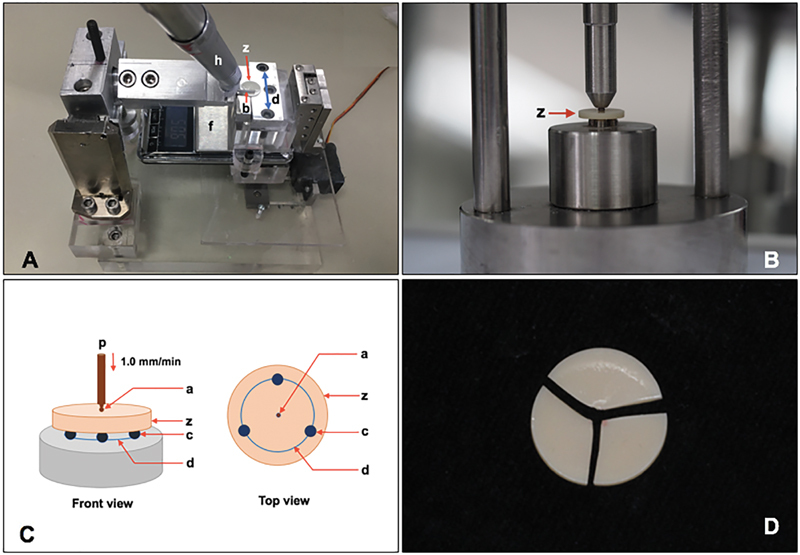
Custom-made machine ( **A** ) was used for controlling the force (f) and direction (d) during finishing and polishing on the surface of zirconia (z) with bur (b) in the fixture mounted hand-piece (h). Biaxial flexural strength was determined by using a piston on three balls apparatus ( **B, C** ) by placing the zirconia disc (z) on three balls (c), which were separately arranged in a circular at 120 degrees apart from each other (d), and loaded vertically (p) with a round end piston (a) at a speed of 1.0 mm/min until fracture. Fracture specimens ( **D** ) were further examined microscopically for analysis of fracture.

All specimens were randomly divided into four groups according to post-sintering surface treatment: AG (as-glazed), FP (finished and polished), FPOG (finished, polished, and overglazed), and FPHT (finished, polished, and heat-treated) groups. The specimens in the AG did not receive any surface treatment. The specimens in the FP were ground by cylinder fine diamond finishing bur (882F, Frank Dental, Gmund, Germany) by an air-rotor with a speed of 400,000 rpm and water coolant. The contact pressure was exactly 50 g and 30 seconds finishing time for each step in a continuous stroke. The horizontal movement was conducted in one direction with the custom-made load and direction-controlled machine with a fixture for holding the grinding handpiece ( Fig. 1A ). The finishing bur was changed to a new one for every single specimen. Then, the specimens were finished with a diamond abrasive bur (Dura green, Shofu, Kyoto, Japan) with a straight handpiece at 20,000 rpm, in continuous strokes and sweeping motions. The polishing procedure was performed by the three-step diamond-impregnated silicone polishing system: coarse, medium, and fine grit (ZilMaster, Shofu). The specimens in the FPOG were ground, finished, and polished similar to those in the FP, and finally overglazed, as previously described. The specimens for the FPHT were finished, polished similarly to the FP, and heat-treated at 910 °C for 1 minute in a ceramic furnace.

### Determination of Biaxial Flexural Strength

The specimens were tested on the piston-on-three-ball apparatus ( Fig. 1B, C ). The testing apparatus comprised three spherical steel balls with a Ø of 4.5 mm, which were arranged in a circular shape with a Ø of 11 mm and separately arranged 120 degrees apart from each other ( Fig. 1C ). The specimens were placed on three spherical balls and pressed against a round end piston of Ø 1.4 mm. Then, the force was induced from a universal testing machine (Lloyd, Leicester, United States) at a crosshead speed of 1.0 mm/min. The load was induced until the zirconia fractured ( Fig. 1D ). The load (Newton [N]) at failure was calculated for biaxial flexural strength (σ, MPa) by using equations 1 2 3 .







Where P is a load at fracture (N), and b is the specimen thickness (mm), υ is Poisson's ratio = 0.35, r _1_ is the radius of support circle (mm), r _2_ is the radius of loaded area (mm), and r _3_ is the radius of the specimen (mm).

### Statistical Analysis

The mean and standard deviation (SD) of σ for each group were compared and analyzed by using ANOVA and post hoc Bonferroni multiple comparisons using statistical software (SPSS version 22, Chicago, Illinois, United States) to determine significant differences in the flexural strength with different post-sintering processes. The result was considered statistically significant at the 95% confidence interval (CI). Weibull analysis was used to determine the reliability of flexural strength and to estimate characteristic strength (σ _o_ ) as well as the Weibull modulus (m) by using Weibull ^++^ statistics (ReliaSoft, Tucson, Arizona, United States) according to equations 4 .



Where P _f_ (σ) is fracture probability, σ is fracture strength, σ _0_ is characteristic strength, and m is Weibull modulus.

### Microscopic Examination

The surface topography and fracture surface of the specimens were evaluated with a scanning electron microscope (SEM, Hitachi, Osaka, Japan). The crystalline phases of zirconia were determined by their relative proportion of microstructures using an X-ray diffractometer (XRD, Advance-Bruker, Ettlinger, Germany). The crystal structures were examined at a diffraction angle (2θ degree) of 20 to 90 degrees with a 0.02 degrees step size per second intervals by using copper k-alpha radiation. The crystalline phase was analyzed by cross-reference with the standards database of powder diffraction and measured the intensity of the peaks using X'Pert Plus software (Philips, Almelo, Netherlands).

## Results

**Fig. 2:**
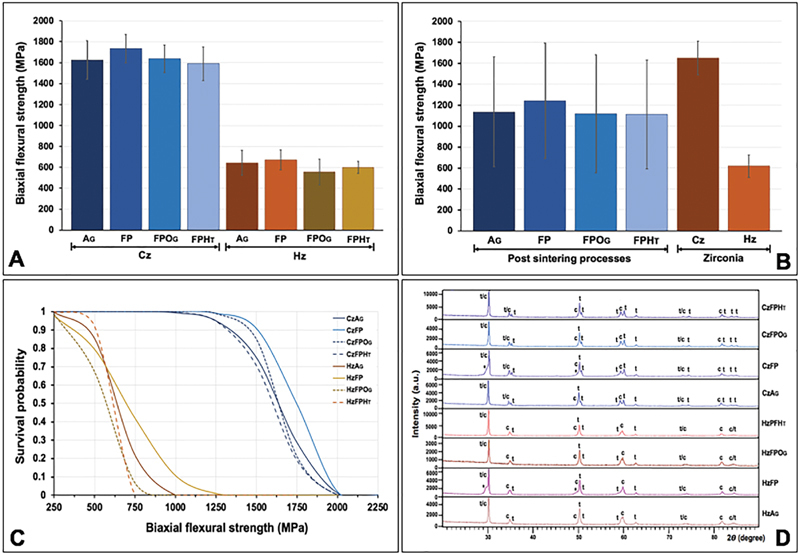
Biaxial flexural strength ( **A, B** ), Weibull survival probability ( **C** ), and X-ray diffraction pattern ( **D** ) of the classical (Cz) and high- translucent zirconia (Hz) upon postprocessing surface treatment with as-glazed, finished and polished, finished, polished and overglazed and finished, polished, and heat-treated techniques.

The mean, SD, 95% confidence interval of σ, σ _o_ , and m for each group are shown in Table 1 and Fig. 2A . ANOVA indicated a statistically significant difference in flexural strength upon postprocessing processes and type of zirconia ( *p* < 0.05), but no interaction effect ( *p* > 0.05) was found ( Table 2 ). The results indicate that the Cz possessed significantly higher flexural strength than the Hz ( *p* < 0.05; Fig. 2B ). The post-sintering processes revealed a statistically significant effect on the flexural strength ( *p* < 0.05). The mean ± SD values of flexural strength upon post-sintering surface treatment with AG, FP, FPOG, and FPHT were 1,134.87 ± 523.19; 1,241.23 ± 552.70; 1,116.64 ± 564.06; and 1,111.68 ± 518.99 MPa, respectively ( Fig. 2B ). Post hoc multiple comparisons showed significant differences in flexural strength upon the FP process to other processes. However, no significant difference in flexural strength was observed among AG, FPOG, and FPHT ( Table 3 ). The post-sintering process with FP significantly enabled flexural strength enhancement for both Cz and Hz but did not affect by other processes. Weibull analysis of the reliability of flexural strength for both Cz and Hz upon different post-sintering processes indicated the “m” varied among groups and indicated their survival probability of the material upon flexural strength ( Table 1 , Fig. 2C ).

**Table 1:** Mean; standard deviation; 95% confidence interval; and characteristic strength, Weibull modulus, and relative tetragonal and cubic phase content (wt.%) of the classical and high translucent zirconia upon postprocessing surface treatment with as-glazed, finished and polished, finished, polished and overglazed and finished, polished and heat-treated techniques

Group	Zirconia	Post-sintering process	*n*	Mean ± SD (95% CI)	σ _o_ (MPa)	m	Relative phase (wt.%)	
							t-phase	c-phase
CzAG	Cz	AG	14	1,626.43 ± 184.38 (1,519.98–1,732.90)	1,709.79	9.51	80.7	19.3
CzFP	Cz	FP	15	1,734.98 ± 136.15 (1,659.59–1,810.38)	1,799.17	12.83	76.2	23.8
CzFPOG	Cz	FPOG	14	1,636.92 ± 130.11 (1,561.80–1,712.05)	1,697.63	14.66	74.3	25.7
CzFPHT	Cz	FPHT	15	1,590.78 ± 161.7 (1,501.22–1,680.35)	1,663.82	10.13	80.4	19.6
HzAGG	Hz	AG	14	643.30 ± 118.59 (574.83–711.78)	695.55	5.59	33.4	66.6
HzFP	Hz	FP	13	671.52 ± 96.77 (613.04–730.01)	782.61	3.28	48.2	51.8
HzFPOG	Hz	FPOG	13	556.33 ± 122.85 (482.09–630.56)	607.01	4.76	47.4	52.6
HzFPHT	Hz	FPHT	14	598.36 ± 57.96 (564.89–631.83)	624.89	11.22	37.1	62.9

Abbreviations: σ _o_ , characteristic strength; AG, as-glazed; FP, finished and polished; FPOG, finished, polished, and overglazed; FPHT, finished, polished, and heat-treated; CI, confidence interval; m, Weibull modulus; Cz, classical translucent zirconia; Hz, high translucent zirconia; SD, standard deviation.

**Table 2:** An analysis of variance of biaxial flexural strength of the different type of zirconias upon different post-sintering processes

Source	SS	df	MS	F	*p* -Value
Corrected model	29,957,150.772	7	4,279,592.967	246.208	0.000
Intercept	143,236,208.018	1	143,236,208.018	8,240.472	0.000
Process	215,967.132	3	71,989.0443	4.142	0.008
Type of zirconia	29,623,593.473	1	29,623,593.47	1,704.265	0.000
Process* type	50,631.187	3	16,877.062	0.971	0.409
Error	1,807,732.092	104	17,382.039		
Total	180,158,723.114	112			
Corrected total	31,764,882.863	111			

Abbreviations: df, degree of freedom; F, F-ratio; MS, mean square; SS, sum of squares.

**Table 3:** Multiple comparisons of biaxial flexural strength of monolithic zirconia after treated surface through different postprocessing surface treatment with as-glazed; finished and polished; finished, polished, and overglazed; and finished, polished, and heat-treated techniques

Group	AG	FP	FPOG	FPHT
AG	1	0.019	1	1
FP		1	0.04	0.002
FPOG			1	1
FPHT				1

Abbreviations: AG, as-glazed; FP, finished and polished; FPOG, finished, polished, and overglazed; FPHTM, finished, polished, and heat-treated techniques.

The XRD analysis of the crystalline contents of the Cz and Hz was illustrated in Table 1 and Fig. 2D . The XRD patterns for both Cz and Hz demonstrated a large amount of t- and c-phase. There was no m-phase observed in both Cz and Hz. The dominant peaks of the t-phase were observed upon the 2θ degree of 30.2, 34.8, 35.34, 50.19, and 59.54 degrees that correlated with the 101-, 002-, 110-, 111-, and 103-crystalline planes, respectively. The dominant peaks of the c-phase were detected at the 2θ degree of 29.9, 34.68, 49.5, and 59.54 degrees, which corresponded to the 111-, 020-, 022-, and 131-crystalline planes, respectively. There were the broad peaks of t-phase at 101-crystalline plane for both CzFP and HzFP, which refer to rhombohedral (r-) or distorted t-phase. The XRD patterns of Cz mostly indicated the t- phase and a minor amount of the c-phase vis versa for Hz.

**Fig. 3:**
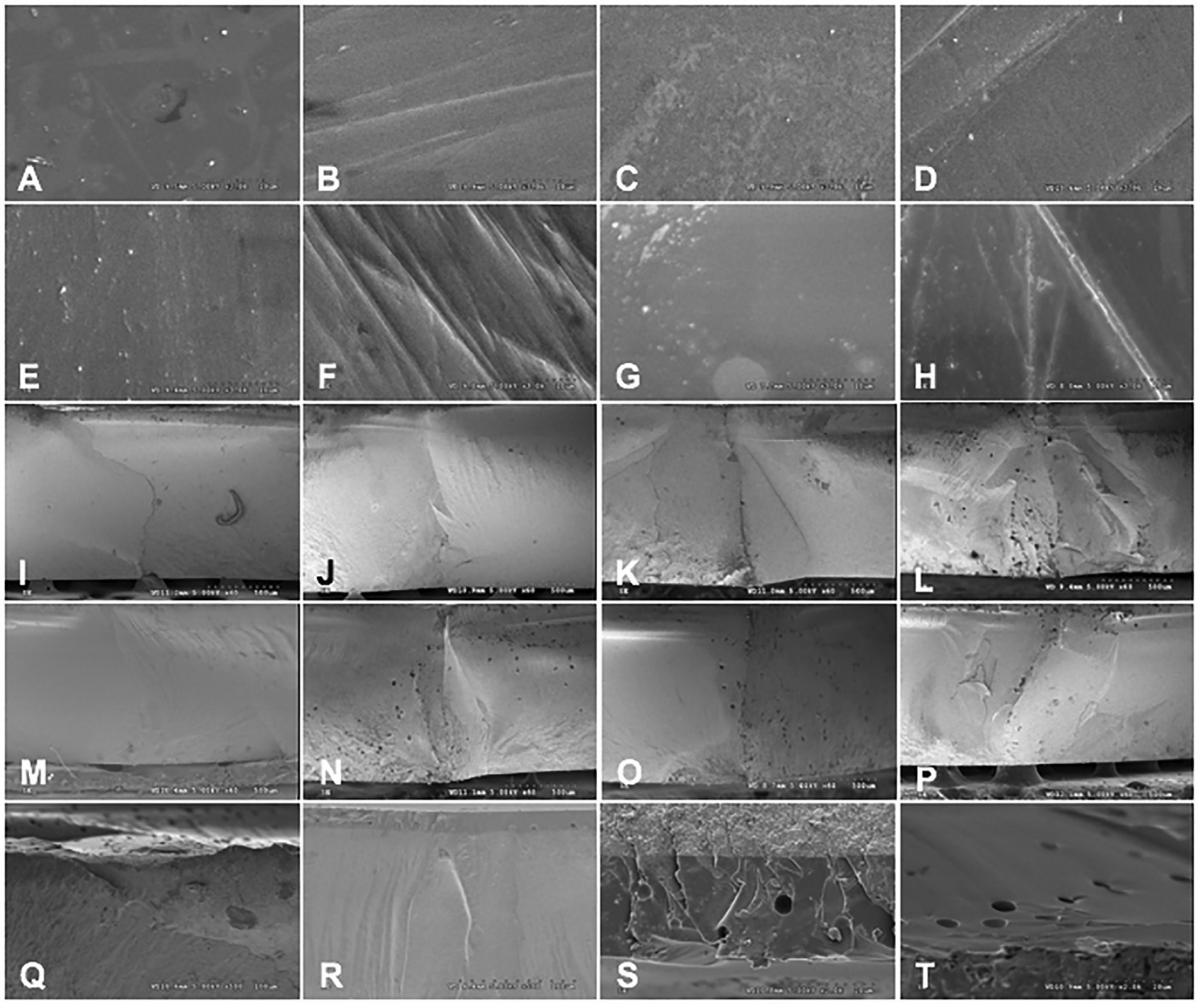
Scanning electron microscope photomicrographs of topographic surfaces ( **A–H** ) (30Kx) and fracture surfaces ( **I–P** (60 × ), and **Q–T** [500 × ]) of the classical ( **A–D, I–L** ) and high translucence zirconia ( **E–H, M–P** ) upon postprocessing treated surface with as-glazed ( **A, E, I, M** ), finished, and polished ( **B, F, J, N** ), finished, polished and overglazed ( **C, G, K, O** ) and finished, polished and heat-treated ( **D, H, L, P** ) techniques. Voids were indicated in the glazed layer in the as-glazed ( **Q, R** ) and overglazed ( **S, T** ) of the classical ( **Q, S** ) and high translucence zirconia ( **R, T** ).

The SEM photomicrographs revealed the irregularities of the surfaces of the CzAG, CzFPOG, HzAG, and HzFPOG due to small particles of glazing material, and some areas which possibly indicated the incomplete adhesion of the glazing materials as well as several voids inside the glazed layer ( Fig. 3A, C, E, G ). The topography of the CzFP, CzFPHT, HzFP, and HzFPHT consisted of scratch lines in one direction, without a distinguished difference ( Fig. 3B, D, F, H ). Meanwhile, the overglazing or FPOG ( Fig. 3C, G ) exhibited a smooth surface rather than a polished surface or FP ( Fig. 3B, F ). The similarity in the crack patterns of Cz, and Hz was revealed. The fracture path originated from the glazed layer and ran through the specimens. The crack propagation demonstrated a straight-line pattern, with sharp flaws which indicated a brittle nature ( Fig. 3I–P ).

## Discussion

This study indicated that post-sintering processes significantly affected the biaxial flexural strength of different types of monolithic zirconia. Therefore, the null hypothesis was rejected for the post-sintering processes and types of zirconia. The grinding, finishing, and polishing procedures were the stepwise method, which was needed to proceed from the coarsest to the finest grit size. The purpose of these procedures was to achieve a smooth, mirror-like surface that provided less susceptibility to bacterial plaque accumulation. 15 The surface adjustment is unavoidable even if the restoration is close to perfect after sintering. The occlusal surface and cervical contour areas must be adjusted clinically during the trial process. 13 14 15 16 A diamond rotary bur was the first step in the zirconia adjustment. A coarse diamond bur has been used in many studies of zirconia surface treatment. 2 5 12 13 15 20 21

Grinding with a coarse diamond bur produced a higher degree of surface roughness than that for the polished surface. The ground zirconia showed significant deterioration in its long-term mechanical properties, which are negatively affected by aging. 5 However, many studies claim that grinding by coarse diamond burs improves the flexural strength because of the transformation toughening mechanism and high content of the m-phase. 20 21 22 23 24 25 Some studies have found no significant correlation between roughness and flexural strength, 15 16 especially when using a small diamond grit size. 5 14 This study used fine grit diamond (38–45 µm in grit size), which also can remove the surface of the zirconia. A small grit-size grinding combined with a proper polishing procedure and coolant may not influence the t- → m-phase transformation because it probably causes a smaller rise in surface temperature while treating the zirconia surface. 25 The ground surface has to be polished to reduce its roughness 13 19 21 and weakening from the grinding of the diamond bur to prevent deleterious effects of low-temperature degradation (LTD). 16 The zirconium dioxide itself is extremely hard even harder than natural teeth. If the contact point is too high, it will cause huge wear and tear on the antagonist natural dentition. 16

The multistep zirconia polishing kit can reduce the zirconia surface by a depth of approximately 3 to 4 µm, which is higher than the coarse-diamond ground-induced transformation layer with a thickness of 0.3 µm. 26 However, the XRD did not detect the m-phase in this study, although the emergence of the t-phase after heat treatment was observed. In the XRD pattern, the finished and polished zirconia had different left hump broad shoulder peaks at 30 and 50 degrees. This could be the r-phase or distorted t-phase. The t-phase or c-phase can change to an r-phase, which can be found as a left hump peak at 30 degrees, as seen in other studies. 18 27 The left hump broad pattern was found in only the FP group of both types of zirconia. The c- → r-phase transformation caused the volume to increase approximately 5.2%, and the t-→ r- transformation caused the volume to increase approximately 3.9%. Hence, the compressive layer of this transformation occurred within the 20 µm layer, 18 and it can occur in both 3Y-TZP and 5Y-PSZ. 11 In many studies, only the r-phase was found for sandblasted or grinding zirconia 11 ; the left hump broad pattern was gone after heat treatment at 1,000°C for 1 hour. 18 This phenomenon was also found in this study, which heat-treated zirconia at 910°C for 1 minute. This indicates that the occurrence of the r-phase leads to a crack-stopping mechanism. Furthermore, the flexural strength of the FP group may be affected by the reduction in the size of flaws; these were still shallow in the specimen, as evident on the SEM. The polishing procedure did not remove all the strength-determining grinding-induced flaws. 25 28 The sequential multistep polishing procedures are still recommended and widely used because of their ability to produce high-gloss surfaces in zirconia comparable to glazed surfaces. 16 22

The gloss finish was also produced by applying glaze material. Flexural strength results in this study were significantly lowered, possibly because of moisture in the glazing mixture and heat from the glaze firing. 17 Indeed, some studies obtained the same result, with the glazing procedure reducing the flexural strength because of the glazing material itself and their manipulation. 17 29 It was found that the mixture of glazing components trapped air bubbles within the glazed layer. The air bubbles inside the glazed layer may represent a trigger point of failure. Moreover, the glass matrix in the mixed glazing paste did not melt or adhere properly to the zirconia, as it does with glass-based ceramics. 2 In areas demanding high esthetics, additional glazing shall be applied to the zirconia because the polishing procedure can decrease its brightness 22 and produce disharmonious color compared to the natural teeth.

Heat treatment can reverse the t- → m-phase transformation when heat is applied at 910°C for 1 minute. 24 29 The opposite was demonstrated in this study, where the amount of m-phase could not be detected, but the r-phase was found. The increase of the t-phase was found in heat-treated zirconia, and the highest t-peak was also found in XRD compared to FP and FTOG. The heat treatment seems to be less affected by Hz, probably because of the lower ability of the Hz to change phase. This result was consistent with that of other studies. 2 14 17 23 24 25 Although the FPOG was subjected to the same firing cycle as FPHT, both procedures exhibited comparable flexural strength, but the FPOG produced a lower level of t-phase, especially in the Cz. Although the flexural strength of the FPHT was the lowest, it was still greater than that designated for monolithic four-unit bridges. The SEM showed the surface irregularities of the FPHT which did not differ from those of the FP, which means that the heat treatment applied in this study did not repair the flaws or porosity of the surface. The LTD or aging of zirconia can occur and leaves the m-phase on its surface, which may weaken the restoration in the long term. Aging may be reduced by heat treatment, which may be helpful for the long-term service life, as found in another study. 11

The Weibull analysis provided the m, σ _o_ , and survival probability. The m in ceramic was used to determine the reliability of the material and the distribution of flaws. A higher m had higher reliability or homogenous distribution of flaws and greater reliability. 30 Flaws in the material were caused by an uneven surface of specimen preparation and processing of the material. 25 Most of the lower m values in this study were found in the Hz group, which may cause by specimen preparation. The σ _o_ was different in each post-sintering process. The FP had the highest σ _o_ , which means that finished and polished zirconia can tolerate more force before it fails and is more durable for long-term use. Moreover, when comparing the σ _o_ of the overglazed and heat-treated group to the as-glazed group in both Cz and Hz, the former was found to be lower than the latter.

This study showed that the group subjected to grinding and then glazing exhibited lower σ _o_ because of the incompatibility between the glazed layer and the zirconia itself. 13 Moreover, the glazed layer diminished over time. An observation showed a loss in the glazed surface due to wear by antagonist natural dentition; in particular, the rough zirconia did not polish well before the glazing application. 22 Hence, in the case of Cz and Hz, the postpolishing glazing procedure and heat treatment are not necessary. This can help reduce the treatment period and obviate any need for complex procedures. The glazing and staining shall be done in a high-esthetic demanding area to provide natural-seeming color to the adjacent teeth and a mirror-like surface finish by glazing or polishing the full-contour zirconia. Nevertheless, the restoration should be checked upon surface treatment during the trial process to ensure no defect exists before cementation. In addition, any grinding adjustment on the surface of cemented zirconia restoration needs to be polished to enhance flexural strength, prevent plaque accumulation, and reduce wear of opposing natural teeth.

## Conclusion

Based on the results of the study, the biaxial flexural strength of the Cz was stronger than that of the Hz. Post-sintering processes affected the flexural strength of monolithic zirconia. The flexural strength of monolithic zirconia can be enhanced through a proper finishing and polishing procedure. Overglazed- and heat-treated processes after finishing and polishing are not necessary because they can reduce the flexural strength of the zirconia. Heat treatment can reverse the phase back to tetragonal but result in a reduction in the flexural strength. However, overglazing shall be done in the esthetic region of restoration to achieve a natural-looking restoration as a gloss surface is also needed for the translucent monolithic zirconia.
